# Medical Association of Georgia

**Published:** 1878-02

**Authors:** 


Medical Association of Georgia.—The annual meeting of our State Medical Association will be held in Atlanta on the third Wednesday, 17th of April.

The committee of arrangements had a meeting last week, and are stirring themselves to have everything in readiness, so as to give our medical brethren a hearty welcome and a good time generally.

Arrangements will be made to lighten the expenses of members by securing half-fare rates on the several railroads, and hotel accommodations at moderate rates.

The time being perhaps the most favorable during the year for physicians to leave their business, and withal the most pleasant season, we shall expect a large attendance.

These yearly reunions of the profession are not only pleasant, but profitable. Coming up from different portions of the State, all, as it were, are linked together in the same brotherhood, and interchange freely their views on subjects connected with the healing art.

The benefits of these meetings are not by any means confined to the medical profession. They constitute a source of advancement in medical knowledge from which public benefits are derived.

Usually a large amount of matter is introduced for the consideration and discussion of members, and we hope there will be no falling off this year. Reports from the various sections, voluntary papers and oral reports of cases afford a rich treat to lovers of medical science.

